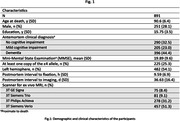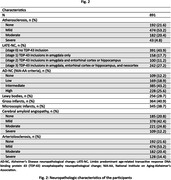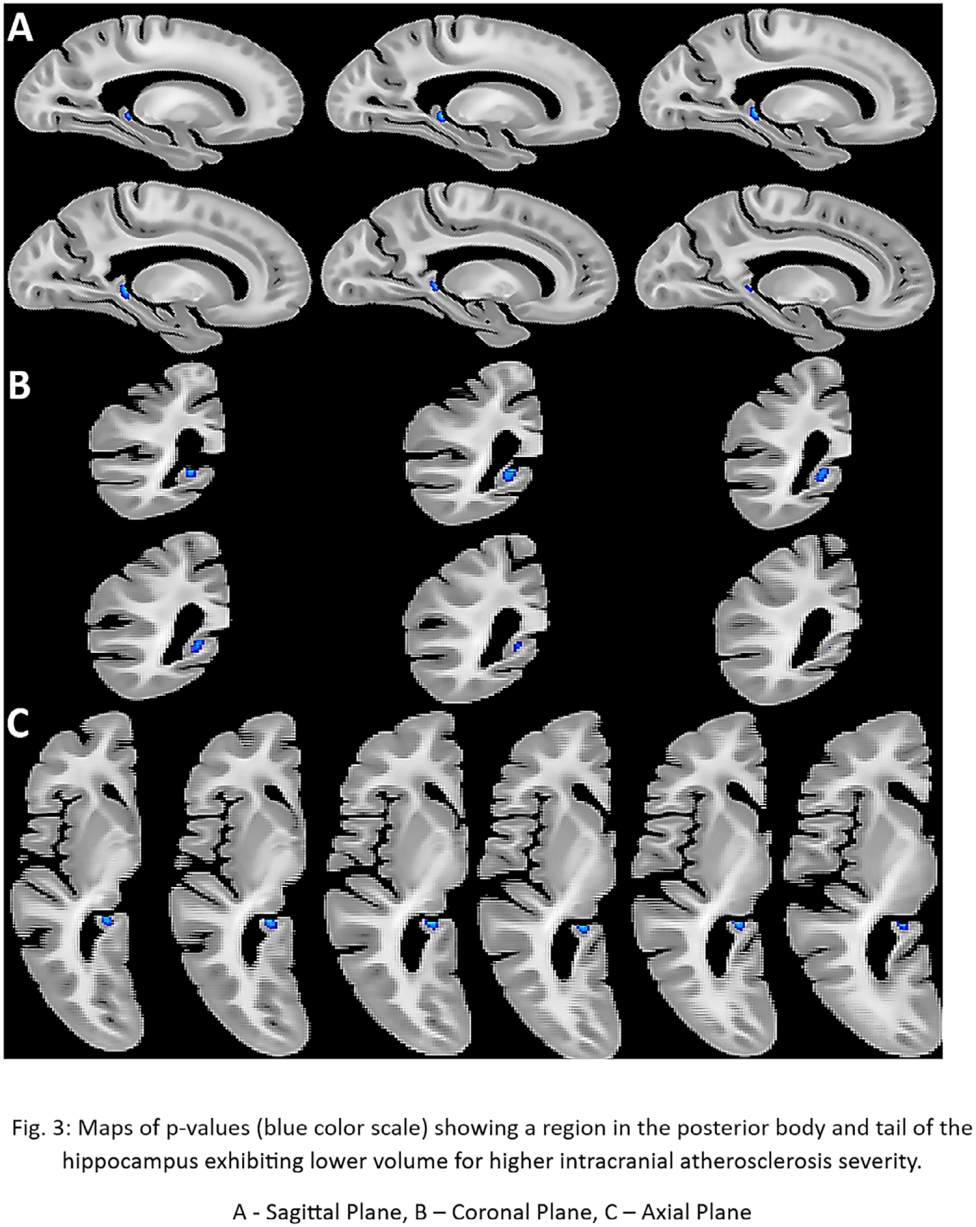# Intracranial atherosclerosis is linked to lower volume in the posterior portion of the hippocampus

**DOI:** 10.1002/alz.087109

**Published:** 2025-01-03

**Authors:** Gulam Mahfuz Chowdhury, Mahir Tazwar, Arnold M Evia, Alifiya Kapasi, Sonal Agrawal, David A. Bennett, Julie A. Schneider, Konstantinos Arfanakis

**Affiliations:** ^1^ Illinois Institute of Technology, Chicago, IL USA; ^2^ Rush University Medical Center, Chicago, IL USA

## Abstract

**Background:**

Intracranial atherosclerosis is a common age‐related neuropathology that has been linked to cognitive decline and dementia and often mixed with Alzheimer’s and other neuropathologies. But the association of atherosclerosis with brain morphometric abnormalities has not been explored. This work combined Deformation‐based morphometry on ex‐vivo MRI with detailed neuropathological examination in a large number of community‐based older adults to investigate the association.

**Method:**

Hemispheres from 891 community‐based older adults from four cohort studies of aging: the Rush Memory and Aging Project, Religious Orders Study, Minority Aging Research Study, and African American Clinical Core of the Rush Alzheimer’s Disease Research Center were imaged *ex‐vivo* on 3T clinical MRI scanners using a multi‐echo spin‐echo sequence with a voxel size = 0.6 × 0.6 × 1.5 mm^3^. All images were non‐linearly registered to an ex vivo brain hemisphere template using ANTs. The logarithm of the Jacobian determinant of the deformation fields was calculated in each voxel and the resulting maps were smoothed using a Gaussian filter with a FWHM = 4mm. All hemispheres underwent detailed neuropathologic examination. The assessed pathologies included atherosclerosis, arteriolosclerosis, cerebral amyloid angiopathy, gross and microscopic infarcts, Alzheimer’s pathology, Lewy bodies, limbic‐predominant age‐related TDP‐43 encephalopathy neuropathological change, and hippocampal sclerosis.

Voxel wise linear regression was used to test the association of atherosclerosis with deformations shown in the smoothed LogJ maps, controlling for other age‐related neuropathologies, demographics (age at death, sex, years of education), postmortem interval to fixation, postmortem interval to imaging, and scanner (Fig. 1,2). The FSL PALM tool with 1000 permutations, threshold‐free cluster enhancement, and tail acceleration was used for the statistical analysis. Associations were considered significant at p<0.05 after family‐wise error rate correction for multiple comparisons.

**Result:**

Voxel‐wise linear regression showed that intracranial atherosclerosis was significantly associated with lower volume in the posterior body and tail of the hippocampus (p<0.05), independently of the effects of other age‐related neurodegenerative and vascular pathologies (Fig. 3). No part of the brain showed significantly higher volume with atherosclerosis.

**Conclusion:**

This work demonstrated that intracranial atherosclerosis is associated with lower volume of the posterior body and tail of the hippocampus. This finding is of great interest due to the important role of the hippocampus in cognition.